# ASIC1a activation induces calcium-dependent apoptosis of BMSCs under conditions that mimic the acidic microenvironment of the degenerated intervertebral disc

**DOI:** 10.1042/BSR20192708

**Published:** 2019-11-13

**Authors:** Feng Cai, Xin Hong, Xiang Tang, Nai-Cheng Liu, Feng Wang, Lei Zhu, Xin-Hui Xie, Zhi-Yang Xie, Xiao-Tao Wu

**Affiliations:** 1 Department of Orthopaedics, The First Affiliated Hospital of Soochow University, Suzhou, China; 2 Department of Spine Surgery, Zhongda Hospital, School of Medicine, Southeast University, Nanjing, China; 3 Department of Neurology, The First Affiliated Hospital of Soochow University, Suzhou, China

**Keywords:** Acid-sensing ion channel 1a, Apoptosis, Bone marrow mesenchymal stem cells, Intervertebral disc degeneration

## Abstract

**Purpose:** In the degenerated intervertebral disc (IVD), matrix acidity challenges transplanted bone marrow mesenchymal stem cells (BMSCs). The Ca^2+^-permeable acid-sensing ion channel 1a (ASIC1a) is responsible for acidosis-mediated tissue injury. The aim of our study was to confirm whether ASIC1a activation induces BMSC apoptosis under conditions that mimic the acidic microenvironment of the degenerated IVD.

**Methods:** ASIC1a expression in rat BMSCs was investigated by real time-PCR, Western blot (WB) and immunofluorescence. The proliferation and apoptosis of BMSCs under acidic conditions were analyzed by MTT and TUNEL assays. Ca^2+^-imaging was used to assess the acid-induced increase in the intracellular Ca^2+^ concentration ([Ca^2+^]i). The activation of calpain and calcineurin was analyzed using specific kits, and WB analysis was performed to detect apoptosis-related proteins. Ultrastructural changes in BMSCs were observed using transmission electron microscopy (TEM).

**Results:** Acid exposure led to the activation of ASIC1a and increased BMSC apoptosis. The Ca^2+^ imaging assay showed a significant increase in the [Ca^2+^]i in response to a solution at pH 6.0. However, BMSC apoptosis and [Ca^2+^]i elevation were alleviated in the presence of an ASIC1a inhibitor. Moreover, ASIC1a mediated the Ca^2+^ influx-induced activation of calpain and calcineurin in BMSCs. WB analysis and TEM revealed mitochondrial apoptosis, which was inhibited by an ASIC1a inhibitor, in BMSCs under acidic conditions.

**Conclusions:** The mimical acidic microenvironment of the degenerated IVD can induce BMSC apoptosis by activating Ca^2+^-permeable ASIC1a. An acid-induced elevation of [Ca^2+^]i in BMSCs leads to the subsequent activation of calpain and calcineurin, further resulting in increased mitochondrial permeability and mitochondrial-mediated apoptosis.

## Introduction

Low back pain (LBP) is considered the most debilitating condition worldwide, and it results in substantial healthcare and socioeconomic consequences [1]. Approximately 50% of the population will experience LBP by the age of 30, and 70% of the population will experience LBP at least once in their lives [2,3]. It has been reported that approximately 20% of annual healthcare costs caused in the U.S.A. are related to LBP [4,5]. One of the most common etiologies of LBP is intervertebral disc degeneration (IVDD) [6].

The intervertebral disc (IVD) consist mainly of three cell types: annulus fibrosus cells (AFCs) in the outer region; nucleus pulposus cells (NPCs) in the inner region; and endplate chondrocytes in the cephalic and caudal endplate (EP) [7]. NPCs reside in gelatinous and hydrated extracellular matrix that consists of proteoglycans, in particular aggrecan [8–10]. The annulus fibrosus (AF) comprises 15–25 concentric rings of highly organized collagen fibers and surrounds the nucleus pulposus (NP) region, providing shape and mechanical tensile strength to the IVD [11–13]. The EP is used to connect the adjacent vertebral bodies and diffuse foundational solutes for metabolic exchange and nutrition transport [13,14]. The loss in number and decline in function of NPCs contributes to IVDD.

Recently, an increasing number of biological therapeutic methods involving the use of growth factors, stem cell transplantation, and gene therapy for preventing or reversing the disc degeneration process have been studied [15–21]. Mesenchymal stem cells (MSCs) have several advantages as seed cells; they offer a wide range of sources, easy accessibility, and the ability of orientable differentiation, which make them an ideal potential cell source for NP regeneration [22]. We previously found that bone marrow mesenchymal stem cell (BMSC) transplantation has the ability to promote the regeneration of degenerated discs in a rabbit model [23]. However, we also discovered that the vitality and biosynthesis of BMSCs is relatively insufficient to meet the requirements of severely degenerated discs. This phenomenon might be caused by the harsh microenvironment of the degenerated disc for both endogenous cells and transplanted seed cells [24].

The microenvironment of the degenerated IVD has distinct and extreme chemical characteristics, including an acidic matrix, high extracellular osmolarity, and reduced nutrition [8,25,26]. Wuertz et al. [27] previously found that matrix acidity is the most challenging chemical condition of the degenerated IVD and potentially weakens cell viability and function. The pH in the normal NP is between 7.0 and 7.2, whereas the values as low as 5.5–5.6 have been reported for seriously degenerated NP tissue removed at surgery [24,28,29]. To our knowledge, the acidic sensing abilities of MSCs in the degenerated disc, as well as the mechanisms that regulate the biological behavior of MSCs in response to extracellular acidity, remain largely uninvestigated.

Acid-sensing ion channels (ASICs) are a sort of voltage-insensitive Na^+^ channels that are activated by extracellular H^+^ and permeable to Na^+^. ASIC proteins are a subfamily of the degenerin/epithelial Na^+^ channel superfamily [30,31]. In mammals, the *ASIC1, ASIC2, ASIC3*, and *ASIC4* genes encode six subunit proteins, including ASIC1a, ASIC1b, ASIC2a, ASIC2b, ASIC3, and ASIC4. Activated ASICs predominantly conduct Na^+^ or K^+^, while homomeric ASIC1a is also Ca^2+^-permeable substantially [32,33].

ASICs are involved in sensing extracellular acidosis and in physiologic and pathologic processes such as nociception, hearing, mechanosensation, sour taste, synaptic plasticity, learning and memory, neuronal injury, and breath control [34]. Recent evidence has indicated that ASIC subunit proteins are expressed not only in the nervous system but also in various species and cell types, including MSCs [35]. Of all the subunits, homomeric ASIC1a was predominantly expressed in BMSCs [35]. The activation or sensitization of Ca^2+^-permeable ASIC1a has been confirmed to be responsible for acidosis-mediated ischemic brain injury caused by Ca^2+^ influx in neurons [36]. Intracellular Ca^2+^([Ca^2+^]i) play a vitally important role in many of the biological behaviors of cells, such as cell division and injury [37]. The redundant accumulation of Ca^2+^ following Ca^2+^-permeable ASIC1a activation leads to articular chondrocyte apoptosis [38].

It has been shown that ASIC1a is expressed in mouse BMSCs [35]. However, it is unknown whether ASIC1a activation leads to Ca^2+^ influx and increases the [Ca^2+^]i in BMSCs. It is also uncertain whether acidosis-mediated ASIC1a activation results in MSC death due to an acidosis-evoked increase in the [Ca^2+^]i in the degenerated intervertebral disc microenvironment. Therefore, the purpose of the present study was to confirm whether ASIC1a activation induces BMSC apoptosis under conditions that mimic the acidic environment of the degenerated disc.

## Methods

### Laboratory animals

All protocols for animal tissues were performed in accordance with relevant guidelines and regulations. Fifteen Sprague–Dawley (SD) rats (Jiangsu Academy of Agricultural Sciences, Nanjing) weighing 100 g were used for cell isolation. All animals were killed by an overdose of pentobarbital sodium.

### Isolation and culture of BMSCs

BMSCs were isolated from SD rats weighing 100 g. The bone marrow of the bilateral femur and tibia was rinsed with 10 ml of phosphate buffered saline (PBS) containing 1000 units of heparin. BMSCs were isolated from rat bone marrow aspirate by centrifugation over Ficoll-Paque PLUS density gradient media (1.077 g/ml, GE Healthcare Life Sciences, Switzerland) for 30 min at room temperature [23]. The isolated BMSCs were harvested and cultured in Dulbecco’s modified Eagle’s medium with low glucose (DMEM-LG; Gibco, U.S.A.) at 37°C in a humidified atmosphere with 5% CO_2_. The culture medium was supplemented with 10% (v/v) fetal bovine serum (FBS; Wisent, Inc., Canada), and 1% penicillin–streptomycin (Gibco, U.S.A.) and was changed every 3 days. When they reached 80–90% confluence, the cultured primary BMSCs were digested with 0.05% trypsin supplemented with 0.02% ethylenediaminetetraacetate (EDTA; Gibco, Carlsbad, CA, U.S.A.) and subcultured as passage 1 (P1).

### Differentiation ability of the isolated BMSCs

Osteogenic differentiation: P3 BMSCs were plated at a density of 2 × 10^4^ cells/cm^2^ in six-well plates. Once 60–70% confluence was achieved, osteogenic differentiation medium was added to the six-well plates. Osteogenic differentiation induction medium was purchased from Cyagen Biosciences Company of China and contained 10% FBS, 0.05 mM ascorbic acid, 10 mM β-glycerophosphate, and 100 nM dexamethasone in DMEM with high glucose. The medium was changed every 3 days. After 4 weeks of induction, 2% Alizarin Red S was used to detect the osteogenic differentiation of the BMSCs by staining for calcium nodules.

Adipogenic differentiation: P3 BMSCs were plated at a density of 2 × 10^4^ cells/cm^2^ in six-well plates. The normal culture medium was replaced with adipogenic differentiation medium when the cells reached 100% confluence. Adipocyte induction medium was purchased from Cyagen Biosciences Company of China and contained 10% FBS, 0.5 mM 3-isobutyl-1-methylxanthine, 10 μg/ml insulin, 200 μM indomethacin, and 1 μM dexamethasone. The medium was changed every 3 days. After 4 weeks of induction, oil red O staining was used to identify adipogenic differentiation.

Chondrogenic differentiation: cell suspension containing 2.5 × 10^5^ BMSCs in a 15 ml centrifuge tube was centrifuged for 5 min at 1500 rpm. Then, a 3D BMSC pellet was obtained after the supernatant was removed. Chondrogenic induction medium was purchased from Cyagen Biosciences Company of China and contained ascorbic acid, 50 mg/ml ITS+Premix, 10 ng/ml transforming growth factor β3 and 10 nM dexamethasone in DMEM with a glucose concentration of 4500 mg/L. The induction medium was changed every 3 days. After 4 weeks, the pellets were obtained, fixed in 10% formalin and cryosectioned at a thickness of 8 μm for alcian blue staining.

### 3-(4,5-Dimethylthiazol-2-yl)-2,5-diphenyltetrazolium bromide assay

3-(4,5-Dimethylthiazol-2-yl)-2,5-diphenyltetrazolium bromide (MTT) assay was used to measure the number of viable cells. P3 BMSCs were plated in 96-well cell culture plates at a density of 1000 cells/well and cultured in a humidified incubator. After specific interventions, 20 μl of MTT (5 mg/ml, Sigma, U.S.A.) was added to each well before incubation at 37°C for 4 h. The medium containing MTT was aspirated, and 150 μl of dimethylsulfoxide (DMSO; Sigma, U.S.A.) was added to the wells prior to incubation at 37°C for 30 min. Finally, the number of viable cells was determined by measuring the optical density (OD) values at 492 nm using a microplate reader (Thermo Scientific, U.S.A.).

### Immunofluorescence staining

BMSCs were washed with PBS and fixed in 4% paraformaldehyde for 15 min at room temperature. The cells were washed with PBS three times and permeabilized for 15 min in 0.2% Triton X-100 solution. The cells were incubated in 5% bovine serum albumin (BSA) for 60 min to block nonspecific binding. Then, the cells were incubated overnight at 4°C with rabbit polyclonal anti-Ki67 (1:400, Abcam, U.K.), anti-CD29 (1:200, Abcam, U.K.), anti-CD90 (1:200, Abcam, U.K.), anti-CD44 (1:200, Abcam, U.K.), and anti-ASIC1a (1:200, Alomone Labs, Israel) antibodies. Next, the cells were incubated in the dark for 2 h with Alexa Fluor 488- or Alexa Fluor 594-conjugated donkey anti-rabbit IgG at room temperature. The nuclei were counterstained with 4′,6-diamidino-2-phenylindole (DAPI) at room temperature for 2 min. The fluorescence was visualized using a fluorescence microscope (FV2000, Olympus, Japan). Negative control was performed following the same procedure using nonspecific rabbit IgG instead of primary antibodies.

### Detection of cell apoptosis

After various treatments, apoptotic BMSCs were detected by the TUNEL assay. BMSCs were fixed in ice-cold 4% paraformaldehyde for 45 min at room temperature after being washed with PBS. The cells were exposed to 0.1% Triton X-100 at 4°C for 2 min, incubated with 50 µl of the TUNEL reaction mixture (5 µl of TdT-enzyme solution + 45 µl of nucleotide mixture solution) in the dark at 37°C for 60 min, and exposed to DAPI for 5 min at room temperature. The fluorescence was visualized using a laser scanning confocal microscope (FV2000, Olympus, Japan). The results were expressed as a percentage of the total cell count.

### ASIC1a-mediated intracellular calcium ([Ca^2+^]i)

As described previously, intracellular calcium imaging was performed to assess the [Ca^2+^]i [24]. Cells were pretreated with a selective blocker of ASIC1a, psalmotoxin-1 (PcTX1, 10 nM; Abcam, U.S.A.), for 2 h. BMSCs on coverslips were washed with D-Hanks solution and incubated with 5 μM Fura-2-AM (Sigma, Ireland) at 37°C in a cell incubator for 1 h and then at room temperature for another 20 min. After incubation, the extracellular Fura-2-AM was removed by washing with D-Hanks solution. The fluorescence intensity was monitored using a laser scanning confocal microscope (Olympus, Japan). Continuous recording was performed upon the addition of a solution at pH 6.0, and the recording lasted for 10 min. The average fluorescence intensity was calculated using LSM 5 Image software.

### Real-time RT-PCR

Total RNA was extracted from cultured BMSCs and fresh cortical tissue (positive control) using the RNAiso Plus (TaKaRa, Japan), and used to immediately synthesise cDNA. Real-time quantitative PCR was performed using a SYBR Green PCR kit (TaKaRa, Japan). Reactions were conducted using a 7300 Real-Time PCR System (Applied Biosystems, Foster City, CA, U.S.A.) in triplicate in 96-well plates with final volumes of 20 μl under standard conditions. The 2^-Δ*C*_t_^ and 2^-ΔΔ*C*_t_^ methods were applied to determine the relative expression levels of *ASIC1*. The housekeeping gene *GAPDH* was used as a reference to normalize PCR data, expressed as the ratio of *ASIC1*-to-*GAPDH*. The primers of *ASIC1* were listed in Table 1.

**Table 1:** The primers of real-time RT-PCR

Gene	Forward (5′–3′)	Reverse (5′–3′)
ASIC1	5′-TGACATTGGTGGTCAAATGG-3′	5′-ATCATGGCTCCCTTCCTCTT-3′
GAPDH	5′-ACATTGTTGCCATCAACGAC-3′	5′-ACGCCAGTAGACTCCACGAC-3′

### Western blot analysis

Cultured BMSCs and fresh cortical tissue (positive control) were lysed in lysis buffer containing a protease inhibitor mixture (Beyotime Biotechnology, China) after being rinsed with ice-cold PBS. Total protein was extracted from five different samples from each group. A bicinchoninic acid quantification kit (Thermo, U.S.A.) was used according to the manufacturer’s instructions to quantify the protein concentration. Equal amounts of cell lysates (20 μg/well) were separated using sodium dodecyl sulfate polyacrylamide gel electrophoresis. The separated proteins were transferred to polyvinylidene fluoride membranes (Millipore, U.S.A.). The membranes were blocked with 5% BSA in Tris-buffered saline containing 0.1% Tween 20 (Sigma, U.S.A.) for 1 h. Then, the membranes were incubated with rabbit anti-ASIC1a (1:200, Alomone Labs, Israel), anti-pBAD (1:1000), anti-tBAD (1:2000), anti-cytochrome *C* (Cyt C, 1:1000), anti-cleaved caspase-9 (1:1000), anti-cleaved caspase-8 (1:1000), anti-cleaved caspase-3 (1:1000), and anti-α-tubulin (1:2500) at 4°C with gentle shaking overnight. All of the above antibodies were obtained from Cell Signaling Technology (U.S.A.), except ASIC1a antibody. Next, the membranes were incubated with a horseradish peroxidase-conjugated secondary antibody (goat anti-rabbit IgG, 1:2500; KeyGEN, China) for 1 h at room temperature. The enhanced chemiluminescence method was used to develop the blots according to the manufacturer’s protocol (Millipore, U.S.A.). Each immunoblot band was quantified by densitometry using a Bio-Rad Calibrated Densitometer.

### Calpain and calcineurin activity assay

Calpain activity was measured using the Calpain Activity Fluorometric Assay Kit from Biovision, U.S.A. according to the manufacturer’s instructions. Cells were cultured at pH 6.0 with or without psalmotoxin-1 (PcTX1) (10 nM) or calpeptin (10 µM) for different times. At each time point, cytosolic proteins without cell membrane contamination and lysosomal proteases were extracted by using extraction buffer. The fluorometric assay was based on the detection of the cleavage of the calpain substrate Ac-LLY-AFC. The fluorescence was sequentially imaged with a confocal imaging system (FV2000, Olympus, Tokyo, Japan), and the mean fluorescence intensity measured at 530 nm was quantitatively analyzed. The Calcineurin Cellular Assay Kit (Ambion, U.S.A.) was used to determine calcineurin phosphatase activity according to the manufacturer’s instructions.

### Transmission electron microscopy

The cells were cultured in acidic solution (pH 6.0) with or without PcTX1 (10 nM), calpeptin (10 µM), or CsA (1 µM) for 24 h. After being washed with D-Hanks solution three times, the cells were fixed immediately in 2.5% glutaraldehyde at 4°C for 2 h and then washed and postfixed in 2% osmium tetroxide in 0.15 M cacodylate buffer for 1 h, washed in distilled water, dehydrated in a graded series of ethanol, and embedded in Epon 618 resin. The coverslips were visualized by transmission electron microscopy (TEM; H-7650, HITACHI, Japan) and observed at an appropriate magnification.

### Inhibitor treatment

PcTX1, a selective blocker of ASIC1a, was used at a concentration of 10 nM. The concentration of 1 and 0.1 nM was used to observe the dose-dependent effect of PcTX1. Calpeptin, a specific inhibitor of calpain, was used at a concentration of 10 µM. Cyclosporine A (CsA), a specific calcineurin inhibitor, was used at a concentration of 1 µM. BMSCs were pretreated with PcTX1, calpeptin or CsA for 2 h for the inhibition experiments.

### Statistical analysis

All experiments in the present study were performed separately at least three times, and all data are shown as the mean ± standard deviation (SD). SPSS 20.0 was used for statistical analysis. One-way analysis of variance (ANOVA) or unpaired Student’s *t*-test was used to assess the differences among the groups, and Dunnett’s test was used for further multiple comparisons. Statistical significance was established at *P*<0.05.

## Result

### Characteristics of BMSCs

**Figure 1:**
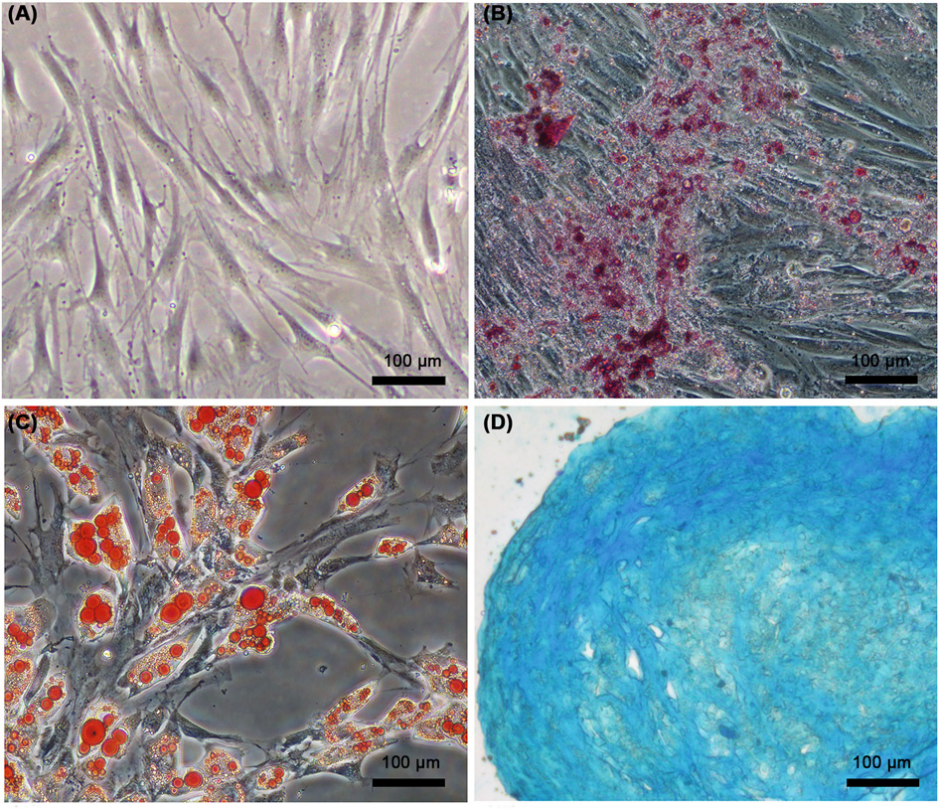
Differentiation of BMSCs Phase contrast Microscope (**A**). Alizarin red S staining showed the calcifying nodules of the extracellular matrix (**B**). Oil Red O staining showed the lipid droplets in cytoplasm (**C**). Alcian Blue staining showed the deposition of proteoglycan in extracellular matrix (**D**).

Isolated rat BMSCs were fibroblast-like, appearing spindle shaped, with granular cytoplasm (Figure 1A). In the present study, P3 cells were used for incubated for 28 days in the presence of specific differentiation agents for osteoblasts, chondrocytes, and adipocytes. Alizarin red S staining showed calcifying nodules in the extracellular matrix (Figure 1B). Differentiation into the adipocytes was demonstrated by staining lipid droplets in the cytoplasm with oil red O (Figure 1C). Alcian blue staining showed the deposition of proteoglycan in the extracellular matrix (Figure 1D).

**Figure 2:**
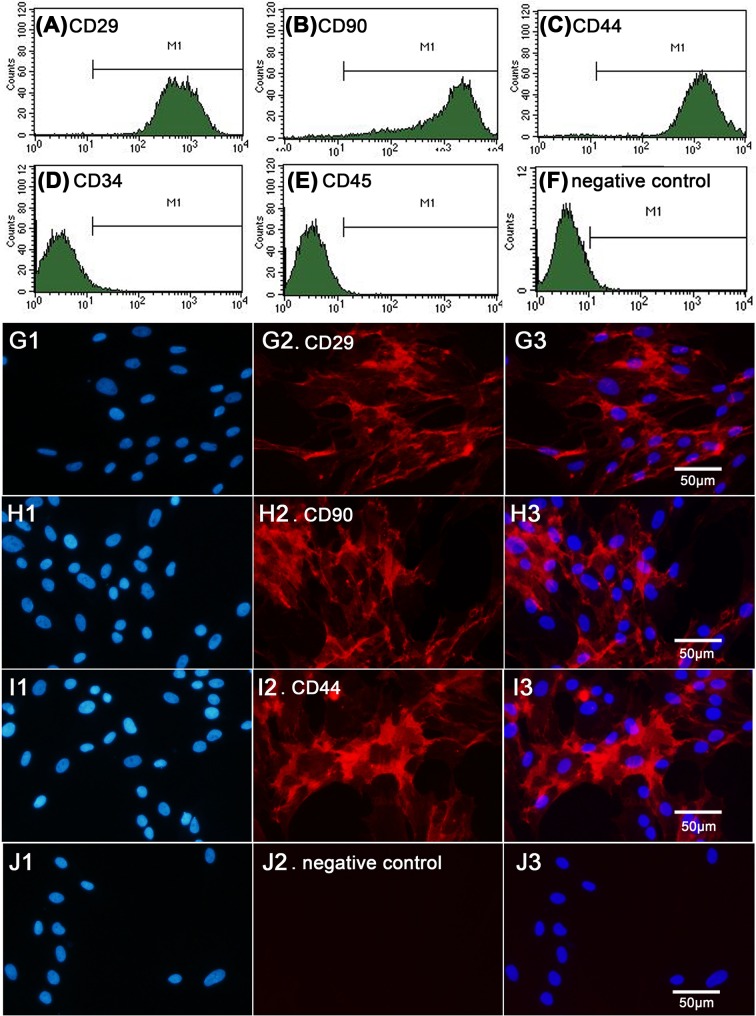
Characterization of cultured rat BMSCs Flow cytometry analysis showed that the isolated BMSCs were positive for CD29, CD90, and CD44, while negative for CD34 and CD45 (**A**–**F**). Immunofluorescence staining showed that the cells expressed characteristic markers of BMSCs, CD29, CD90, and CD44 (**G**–**J**).

Flow cytometry analysis showed that the isolated cells were positive for CD29, CD90, and CD44 but were negative for CD34 and CD45 (Figure 2A–F). In addition, immunofluorescence staining showed that the cells expressed characteristic markers of BMSCs, namely, CD29, CD90, and CD44 (Figure 2G–J).

### The expression of ASIC1a in BMSCs

**Figure 3:**
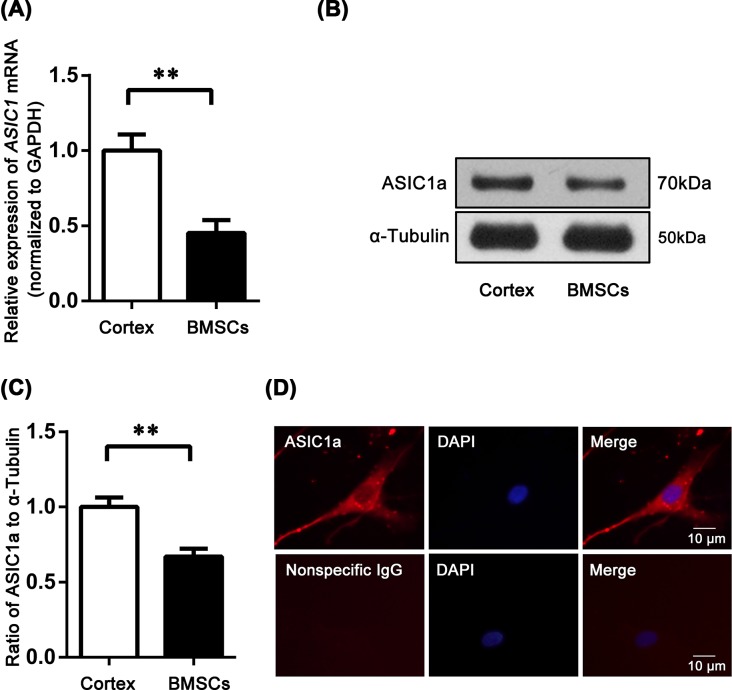
The expression of ASIC1a in BMSCs Gene expression was measured using real-time RT-PCR, and mRNA levels of *ASIC1* in the rat cortex (positive control) were normalized to 1 (**A**). Western blot analysis showed the expression of ASIC1a in BMSCs was lower than that in cortex (**B,C**). ASIC1a was found in the cytoplasm and cytomembrane of BMSCs (**D**). Negative controls with nonspecific rabbit IgG gave no signal. **P<0.01, *n*=5.

Figure 3A showed the expression of *ASIC1* mRNA in BMSCs and rat cortex (positive control). *ASIC1* mRNA expression in BMSCs was lower than in cortex. Similarly, Western blot (WB) analysis showed the expression of ASIC1a in BMSCs was lower than in cortex (Figure 3B,C). As shown in Figure 3D, ASIC1a expression was determined using immunofluoresence analysis. The results showed that ASIC1a was expressed in rat BMSCs. ASIC1a was found in the cytoplasm and cytomembranes of BMSCs. Negative controls with nonspecific rabbit IgG exhibited no signal.

### Analysis of the viability and proliferation of BMSCs under acidic conditions

**Figure 4:**
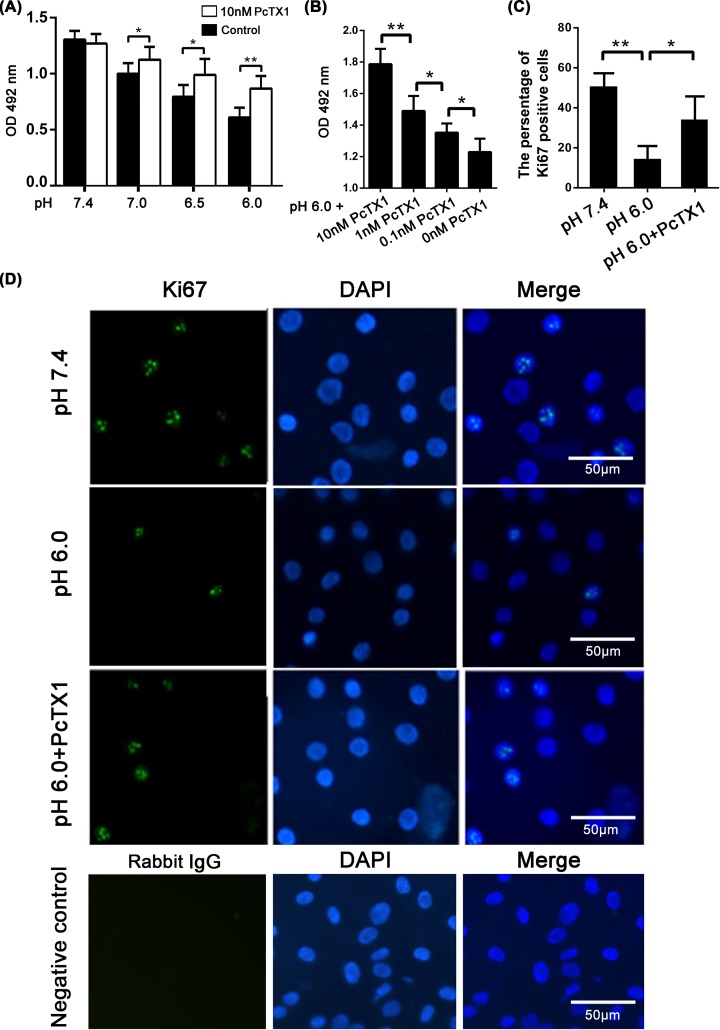
Viability and proliferation analysis of BMSCs in acid condition MTT analysis showed that cell viability was significantly decreased at pH 7.0, 6.5, and 6.0 than that at pH 7.4. However, the cell viability partly recoverd in acid with the presence of PcTX1 (**A**). PcTX1 treatment showed a dose-dependent effect in attenuating phenotypes induced by pH 6.0 (**B**). Immunofluorescence staining showed that Ki67 positive cells significantly decreased at pH 6.0, when compared with pH 7.4. While, PcTX1 treatment could increase the number of Ki67 positive cells cultured in acid condition (**C,D**). *P<0.05, **P<0.01, *n*=5.

The effect of acidic conditions on the viability of BMSCs was evaluated by the MTT assay. As shown in Figure 4A, cell viability was significantly decreased at pH 7.0, 6.5, and 6.0 compared with pH 7.4. However, cell viability partly recovered under acidic conditions in the presence of PcTX1. PcTX1 treatment showed a dose-dependent effect in attenuating phenotypes induced by pH 6.0 (Figure 4B).

To further validate the effect of acidic conditions on the proliferation of BMSCs, we next performed Ki67 immunofluorescence staining. The percentage of Ki67-positive cells significantly decreased at pH 6.0 compared with pH 7.4. PcTX1 treatment increased the number of Ki67-positive cells under acidic conditions (Figure 4C,D).

### Activation of ASIC1a-mediated acid-induced BMSC apoptosis

**Figure 5:**
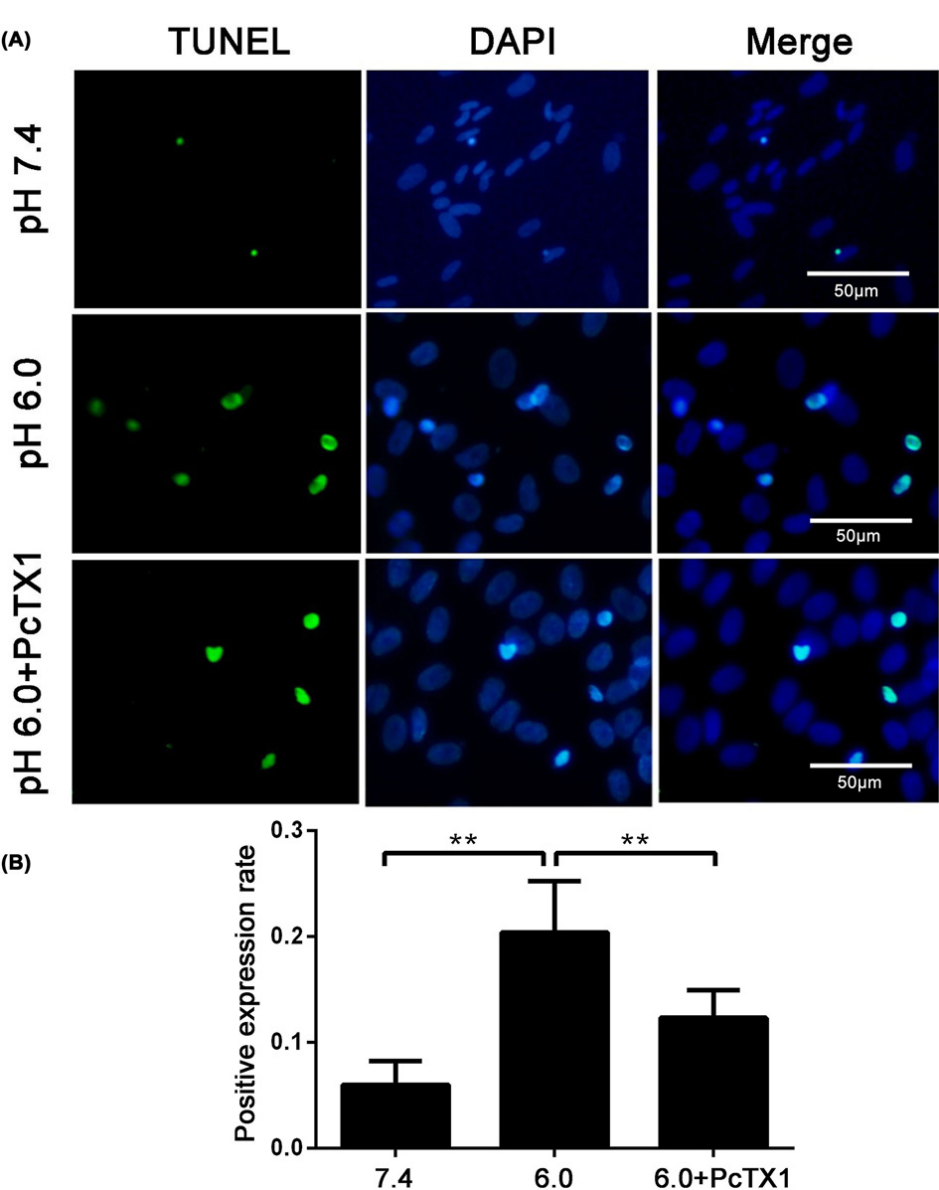
Apoptosis of BMSCs in acid condition TUNEL assay showed that acid exposure significantly induced BMSCs apoptosis, which was inhibited by PcTX1 (**AB**). **P<0.01, *n*=5.

To determine whether extracellular acidosis is involved in BMSC apoptosis, the percentage of apoptotic cells was detected after culture at pH 6.0 and 7.4 for 24 h using a TUNEL assay. In Figure 5, the result obtained from TUNEL staining indicated that the percentage of TUNEL-positive cells was markedly higher at pH 6.0 than at pH 7.4 (*P*<0.01, *n*=5). However, acid-induced BMSC apoptosis was significantly suppressed by the ASIC1a inhibitor PcTX1 (*P*<0.05, *n*=5).

### Acid-induced increase in the [Ca^2+^]i in BMSCs was attenuated by an ASIC1a inhibitor

**Figure 6:**
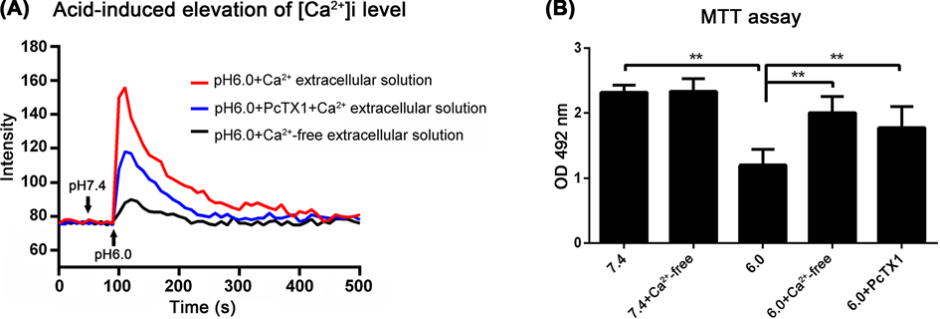
ASIC1a-mediated BMSCs death rely on Ca^2+^ influx Ca^2+^-imaging assay showed a significant increase in [Ca^2+^]i in response to pH 6.0 solution containing Ca^2+^ (**A**). MTT assay showed that ASIC1a blockade with PcTX1 and Ca^2+^-free extracellular solution both increased BMSCs survival in acid (**B**). **P<0.01, *n*=5.

To explore the underlying mechanisms of ASIC1a-mediated BMSC damage, we further detected the [Ca^2+^]i under different conditions (Figure 6A). After exposure to extracellular acidic conditions, Fura-2-AM fluorescence was used for Ca^2+^ imaging to detect the [Ca^2+^]i in BMSCs. Voltage-gated Ca^2+^ channels were inhibited with specific blockers to prevent the secondary activation of these channels. The results of the Ca^2+^ imaging assay revealed a significant increase in the [Ca^2+^]i in response to a Ca^2+^-containing solution of pH 6.0. However, such [Ca^2+^]i increases were almost completely blocked by PcTX1, and this observation demonstrated that a lower pH was able to activate Ca^2+^-permeable ASIC1a in BMSCs and confirmed the essential role of ASIC1a in mediating acid-induced Ca^2+^ signaling in BMSCs.

To further investigate whether Ca^2+^ entry through ASIC1a channels is largely responsible for acid-mediated BMSC death. We cultured BMSCs under acidic conditions (pH 6.0) for 12 h in the presence of a normal or Ca^2+^-free extracellular solution. ASIC1a blockade with PcTX1 and the Ca^2+^-free extracellular solution both increased BMSC survival under acidic conditions (*P*<0.01, *n*=5 both) (Figure 6B). These data indicated that the acid-induced elevation of the [Ca^2+^]i via the activation of ASIC1a is involved in acid-induced BMSC apoptosis.

### ASIC1a activation is essential for the Ca^2+^-mediated activation of calpain/calcineurin in BMSCs

**Figure 7:**
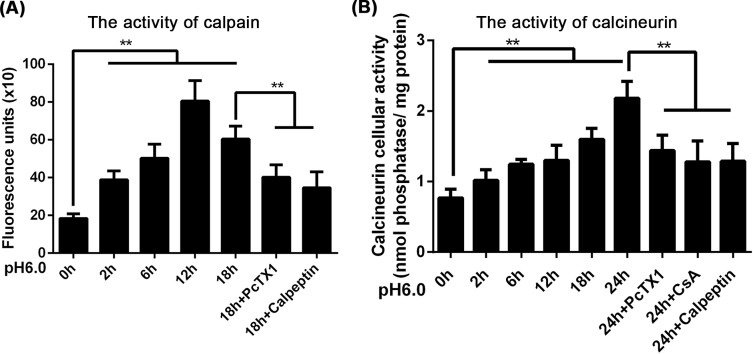
ASIC1a activation is essential for Ca^2+^-mediated activation of calpain/calcineurin in BMSCs Calpain was activated after acidic solution treatment, and its activity reached a maximum at 12 h. The increase in calpain activity was inhibited by PcTX1 or a specific inhibitor of calpain, calpeptin (**A**). Extracellular acidic solution caused a time-dependent increase in calcineurin activity in BMSCs using a calcineurin activity assay kit. The increase in calcineurin activity was inhibited by PcTX1, calpeptin and a specific calcineurin inhibitor, cyclosporine A (**B**). **P<0.01, *n*=5.

Considering that Ca^2+^ is a powerful secondary messenger that affects a number of calcium sensors, we further detected the activity of a Ca^2+^-dependent protease. As shown in Figure 7A, the specific Ca^2+^-dependent protease calpain was activated after acidic solution treatment, and its activity peaked at 12 h (*P*<0.01, *n*=5). The increase in calpain activity was inhibited by PcTX1 and a specific inhibitor of calpain, calpeptin (*P*<0.01, *n*=5) (Figure 7A).

Calcineurin is a cytosolic phosphatase that is activated by calpain and is involved in cell apoptosis. We found that extracellular acidic solution caused a time-dependent increase in calcineurin activity in BMSCs using a calcineurin activity assay kit. The increase in calcineurin activity was inhibited by PcTX1, calpeptin and a specific calcineurin inhibitor, cyclosporine A (*P*<0.01, *n*=5) (Figure 7B). These results indicated that Ca^2+^ induced calpain activation further activated calcineurin.

### The Ca^2+^-dependent signaling pathway results in mitochondrial apoptosis in BMSCs under acidic conditions

**Figure 8:**
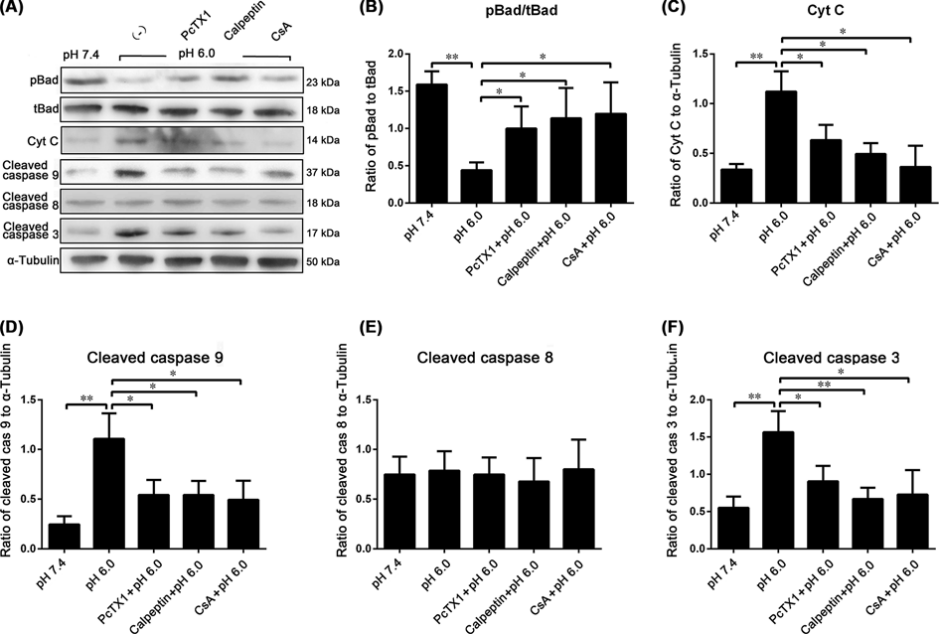
Apoptosis of BMSCs in acid WB showed the apoptosis-related proteins expression of BMSCs in acid (**A**). Bad dephosphorylation increased, which resulted in the decreased expression of pBad. PcTX1, calpeptin, and CsA partly attenuated these effects (**B**). In pH 6.0, the expression of Cyt C, cleaved caspase-9 and caspase-3 increased significantly, which was inhibited by PcTX1, calpeptin and CsA (**C,D,F**). In pH 6.0, the expression of cleaved caspase-8 had no significant change (**E**). *P<0.05, **P<0.01, *n*=5.

The WB results showed an increase in BAD dephosphorylation, which resulted in the decreased expression of pBAD (*P*<0.01). PcTX1, calpeptin and CsA partly attenuated these effects (*P*<0.05, *P*<0.05, *P*<0.05, respectively). At pH 6.0, the expression of Cyt C, cleaved caspase-9 and caspase-3 increased significantly (*P*<0.01, *P*<0.01, *P*<0.01, respectively), and these increases were inhibited by PcTX1, calpeptin and CsA (Figure 8).

**Figure 9:**
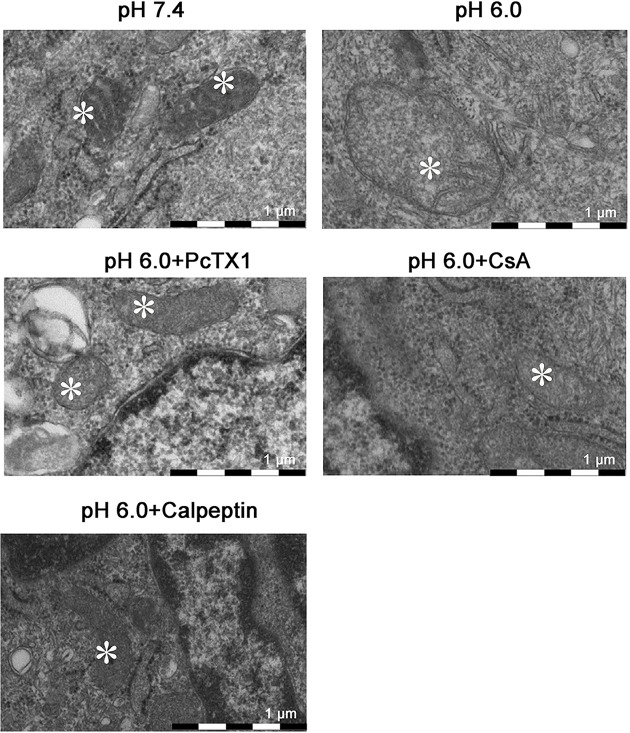
Ultrastructural changes in BMSCs The swelling of mitochondria (asterisk) and reduction or disappearance of mitochondrial crest were observed in BMSCs treated with pH 6.0 solution by TEM. PcTX1, calpeptin, and CsA partly attenuated these effects.

On the other hand, electron microscopy was used to observe the acid-induced ultrastructural changes in BMSCs. Mitochondrial swelling and the reduction or disappearance of the mitochondrial crest were observed in BMSCs under acidic conditions (pH 6.0). PcTX1, calpeptin, and CsA partly attenuated these effects (Figure 9).

## Discussion

IVDs are the largest avascular tissues in the human body, and NPCs consume glucose and produce lactic acid at a relatively high rate, which leads to the accumulation of lactate and the strong acidification of the microenvironment, especially during the process of disc degeneration [27,33]. Although we have found that BMSC transplantation can promote the regeneration of degenerative discs in a rabbit model [23], the vitality of BMSCs is relatively limited in severely degenerative discs.

**Figure 10:**
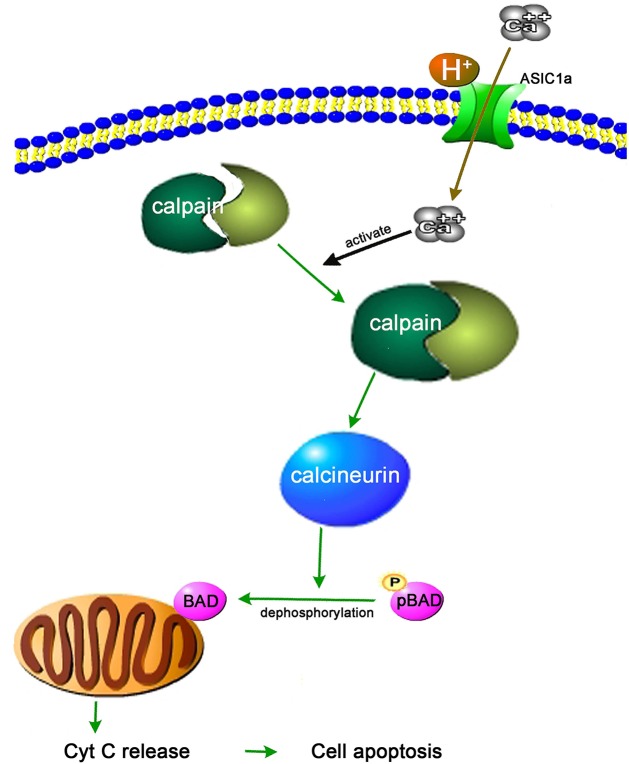
Schematic diagram of the underlying mechanism of ASIC1a-mediated BMSCs apoptosis Arrows indicate enhancement.

In the current study, we found that an acidic environment has a significant negative effect on the viability of BMSCs and that the acid-induced elevation of the [Ca^2+^]i via the activation of ASIC1a is involved in acid-induced BMSC apoptosis. Ca^2+^ is a second messenger in many signaling pathways. To explore the underlying mechanisms of ASIC1a-mediated BMSC damage, we further detected the activity of Ca^2+^-dependent proteases. We found that the acid-induced elevation of the [Ca^2+^]i leads to the Ca^2+^-mediated activation of calpain and calcineurin in BMSCs. Activated calcineurin can promote pBAD dephosphorylation. Dephosphorylated BAD increases mitochondrial permeability and promotes the release of Cyt C from mitochondria (Figure 10). Thus, acid-induced BMSC injury is partly mediated by the mitochondrial apoptosis pathway.

The results of the present study demonstrated that ASIC1a is expressed in rat BMSCs and is activated under acidic conditions. This result is consistent with that of a previous report showing the functional expression of ASIC1a in mouse BMSCs [35]. Previously, we reported that ASIC1a is expressed in human NPCs and can be activated by extracellular acidification, which is involved in NPC acidic injury [24]. Acidosis also exhibits an apoptotic effect on other cells, such as glioma cells, neurons, and articular chondrocytes [39–42]. Although many researchers have focused on BMSC transplantation for the treatment of intervertebral disc degeneration, cell survival after transplantation into acidic environments is poorly understood. Thus, in the present study, we explored the effect of an acidic environment on BMSCs and showed that an acidic environment has a significant pro-apoptotic effect on BMSCs. We also found that acid-induced BMSC apoptosis is potently inhibited by the ASIC1a selective inhibitor PcTX1. The above results suggest that ASIC1a serves as a sensor for extracellular acidosis and mediates the biological response of BMSCs to the acidic environment of the degenerated intervertebral disc.

The mechanisms of ASIC1a-mediated BMSC apoptosis are not clear at this time. Our previous study suggested that the acid-induced elevation of the [Ca^2+^]i via the activation of ASIC1a might be involved in NPC apoptosis. In the present study, we found a similar situation in BMSCs. Ca^2+^ is a powerful secondary messenger that affects a number of calcium sensors, including calpain, a Ca^2+^-dependent cysteine protease, and calcineurin, a Ca^2+^/calmodulin-dependent protein phosphatase. We found that the acid-induced elevation of the [Ca^2+^]i in BMSCs leads to the subsequent activation of calpain and calcineurin. Calpain contains 2 catalytic subdomains, namely, IIa and IIb, that are separated by a deep crevice. The binding of Ca^2+^ and phospholipids to calpain initiates a series of structural movements that results in the close association of IIa and IIb to form a functional catalytic site. The functional catalytic site of calpain induces the proteolytic cleavage of calcineurin. Thus, calpain can directly regulate calcineurin activity through proteolysis. The activation of calcineurin increases phosphatase activity, which promotes the caspase-mediated death of cells, including neuronal cells, chondrocytes and NPCs.

A previous study indicated that the biological behavior of NP-MSC can be inhibited by acidic conditions during intervertebral disc degeneration, but the detailed mechanism was not clearly stated [43]. In the present study, we further showed that calcineurin dephosphorylates BAD and causes Ca^2+^-induced apoptosis. Cyt C released from mitochondria activates caspases and mediates intrinsic pathway apoptosis.

However, there are some limitations to the present study. First, the study was conducted in 2D culture medium. We should conduct the experiments in 3D culture medium to simulate the nature of the IVD. Second, the present study showed that ASIC1a is expressed and functions in rat BMSCs, but the other subunits have not yet been studied. A previous study indicated that ASIC3 was expressed in BMSCs, but only 8% of cells showed typical ASIC3 type whole cell current [35]. ASIC3 inhibitor did not influence cell proliferation or death at different pH values [44]. Importantly, inhibition of ASIC3 prevented the acidic pH induced proinflammatory and pain-related phenotype in NPCs [44]. Also, a recent study showed that ASIC3 may serve as a pH sensor in synoviocytes and may be important for the release of hyaluronan within joint tissue [45]. Thus the authors speculate that ASIC3 may be involved in inflammatory reaction and matrix metabolism in intervertebral disc rather than cell apoptosis. In future research, the present group will also focus on the role of ASIC3 on matrix metabolism and immunoregulation of BMSC in intervertebral disc microenvironment.

## Conclusion

Although BMSC transplantation can promote the regeneration of degenerative discs, the vitality of BMSCs is relatively limited in the harsh microenvironment of degenerative discs. Conditions that mimic the acidic microenvironment of the degenerated intervertebral disc are able to activate Ca^2+^-permeable ASIC1a in BMSCs and confirm the essential role of acid-induced BMSC apoptosis. The acid-induced elevation of the [Ca^2+^]i in BMSCs leads to the subsequent activation of calpain and calcineurin, further resulting in increased mitochondrial permeability and mitochondrial-mediated apoptosis. The present study suggests that ASIC1a may be a target for promoting the adaptation of BMSCs to the harsh microenvironment of the degenerated disc.

## Abbreviations

AFC: annulus fibrosus cell
ASIC: acid-sensing ion channel
BMSC: bone marrow mesenchymal stem cell
BSA: bovine serum albumin
DAPI: 4′,6-diamidino-2-phenylindole
FBS: fetal bovine serum
IVD: intervertebral disc
IVDD: intervertebral disc degeneration
LBP: low back pain
MSC: mesenchymal stem cell
NPC: nucleus pulposus cell
PBS: phosphate buffered saline
TEM: transmission electron microscopy
WB: Western blot
